# Universal Stress Proteins Are Important for Oxidative and Acid Stress Resistance and Growth of *Listeria monocytogenes* EGD-e *In Vitro* and *In Vivo*

**DOI:** 10.1371/journal.pone.0024965

**Published:** 2011-09-30

**Authors:** Christa Seifart Gomes, Benjamin Izar, Farhad Pazan, Walid Mohamed, Mobarak Abu Mraheil, Krishnendu Mukherjee, André Billion, Yair Aharonowitz, Trinad Chakraborty, Torsten Hain

**Affiliations:** 1 Institute of Medical Microbiology, Justus-Liebig-University, Giessen, Germany; 2 Institute of Phytopathology and Applied Zoology, Justus-Liebig-University, Giessen, Germany; 3 Department of Molecular Microbiology and Biotechnology, Tel Aviv University, Tel Aviv, Israel; Fundació Institut Germans Trias i Pujol; Universitat Autònoma de Barcelona CibeRES, Spain

## Abstract

**Background:**

Pathogenic bacteria maintain a multifaceted apparatus to resist damage caused by external stimuli. As part of this, the universal stress protein A (UspA) and its homologues, initially discovered in *Escherichia coli* K-12 were shown to possess an important role in stress resistance and growth in several bacterial species.

**Methods and Findings:**

We conducted a study to assess the role of three homologous proteins containing the UspA domain in the facultative intracellular human pathogen *Listeria monocytogenes* under different stress conditions. The growth properties of three UspA deletion mutants (Δ*lmo0515*, Δ*lmo1580* and Δ*lmo2673*) were examined either following challenge with a sublethal concentration of hydrogen peroxide or under acidic conditions. We also examined their ability for intracellular survival within murine macrophages. Virulence and growth of *usp* mutants were further characterized in invertebrate and vertebrate infection models.

Tolerance to acidic stress was clearly reduced in Δ*lmo1580* and Δ*lmo0515*, while oxidative stress dramatically diminished growth in all mutants. Survival within macrophages was significantly decreased in Δ*lmo1580* and Δ*lmo2673* as compared to the wild-type strain. Viability of infected *Galleria mellonella larvae* was markedly higher when injected with Δ*lmo1580* or Δ*lmo2673* as compared to wild-type strain inoculation, indicating impaired virulence of bacteria lacking these *usp* genes. Finally, we observed severely restricted growth of all chromosomal deletion mutants in mice livers and spleens as compared to the load of wild-type bacteria following infection.

**Conclusion:**

This work provides distinct evidence that universal stress proteins are strongly involved in listerial stress response and survival under both *in vitro* and *in vivo* growth conditions.

## Introduction

Universal stress proteins (Usps) comprise a group of proteins induced by different stress conditions and are found in numerous prokaryotic as well as eukaryotic organisms [1], [2]. Among these, universal stress protein A (UspA) of *Escherichia coli* K-12 is best characterized and found to be highly expressed in response to heat, substrate starvation, exposure to antimicrobial agents and oxidative stress [1].

Subsequently, additional Usps have been described for *E. coli* K-12 and several other bacterial species, including *Haemophilus influenza* [3], [4], *Mycobacterium smegmatis* [5], *Mycobacterium tuberculosis* [2], [6], *Pseudomonas aeruginosa* [7]–[9], *Porphyromonas gingivialis* [10], *Shigella sonnei* [11], *Salmonella typhimurium* [12]–[14] and *Lactobacillus plantarum* [15], [16].

The majority of *usp* genes are monocistronically expressed, and different transcription factors, such as σ^32^, σ^70^ and σ^E^ promote transcription of Usps [1].

The significance of Usps in the model pathogen *Listeria monocytogenes* is presently unknown. *L. monocytogenes* is a ubiquitously occuring gram-positive, facultative intracellular bacterium that causes food-borne infections, which mainly affect pregnant women, newborns, elderly and immunocompromised patients [17], [18]. *L. monocytogenes* possesses the remarkable ability to grow under a wide range of temperatures, pH conditions and high osmolarity, allowing the pathogen to survive in nature, food preservation methods as well as in infected host cells [18].

Here we present first evidence that UspA domain containing stress proteins are of importance for *L. monocytogenes* to survive under different stress conditions both *in vitro* and *in vivo*.

## Materials and Methods

### Bacterial strains, plasmids and culture conditions

All bacterial strains and plasmids used in this study are listed in Table 1.

**Table 1:** Bacterial strains and plasmids used in this study.

Strain or plasmid	Description	Reference or source
*L. monocytogenes* EGD-e	wild-type	[19]
DH10β	electrocompetent	Life technologies
Δ*lmo*0515	*lmo*0515 deletion (379 nt) strain of EGD-e	This study
Δ*lmo*1580	*lmo*1580 deletion (409 nt) strain of EGD-e	This study
Δ*lmo*2673	*lmo*2673 deletion (471 nt) strain of EGD-e	This study
Δ*lmo*0515Δ*lmo*1580Δ*lmo*2673	*lmo*0515, *lmo*1580 and *lmo*2673 deletion strain of EGD-e	This study
Δ*lmo*0515+pPL2*lmo*0515	complementation of *lmo0515* deletion strain of EGD-e	This study
Δ*lmo*1580+pPL2*lmo*1580	complementation of *lmo1580* deletion strain of EGD-e	This study
Δ*lmo*2673+pPL2*lmo*2673	complementation of *lmo2673* deletion strain of EGD-e	This study
pPL2	site specific phage integration vector	[23]
pCR2.1-TOPO®	single 3′-thymidine (T) overhangs for TA Cloning® 3.9 kb	Life technologies
pAUL-A	temperature sensitive shuttle vector 9.2 kb	[21]

The wild type strain *L. monocytogenes* EGD-e serotype 1/2a [19] and its isogenic *usp* deletion mutants or complemented strains were cultivated aerobically in Brain Heart Infusion medium (BHI; Difco) at 37°C or on BHI agar plates at 37°C.

*E. coli* strain (DH10β^M^) was grown in Luria-Bertani broth or LB agar plates at 37°C. When required, antibiotics were added to the following concentrations: erythromycin (Sigma), 300 µg/ml for *E. coli* and 5 or 10 µg/ml for *Listeria*; ampicillin (Sigma), 50 µg/ml for *E. coli* and 200 µg/ml for *Listeria*.

### Construction of chromosomal *usp* deletion mutants

Generation of the *usp* in frame deletion mutants was done as previously described [20]–[22]. Briefly, the flanking regions of each *usp* gene were amplified by PCR (*Taq* polymerase; Fermentas) using primer pairs 1 and 2b for the 5′ flanking region and 3b and 4 for the 3′ flanking region (Table 2), respectively. The resulting PCR products were fused to each other by using primers 1 and 4 in a second PCR reaction with Taq polymerase and the product was cloned into pCR2.1-TOPO®.

**Table 2:** Primers used in this study.

Name	5′-3′ Sequence
lmo0515-1	GCATTGCCACAAACTGGTGA
lmo0515-2b	AAGTGTCGCTGCTACAAGAATGCGATG
lmo0515-3b	CTTGTAGCAGCGACACTTTAATAGCTA
lmo0515-4	TTTTGGGGCTGATCCTACGC
lmo0515-5	ATTATTGTGAATTCATTAG
lmo0515-6	TACTTCGATCCATTTTGAT
lmo0515-7	CGACAGTGGACATGTTGATC
lmo0515-8	ACCCAATTTGGTCATGCGAT
lmo1580-1	GGGTCATTGCCACCCTATT
lmo1580-2b	ACATCGCAAGAATAATTCCTCCAATCAAAA
lmo1580-3b	AGGAATTATTCTTGCGATGTTCTTGTAGTTCG
lmo1580-4	GTTTTCGTTGACCGTATCCA
lmo1580-5	CTAAATCACTCTCCTCGTTA
lmo1580-6b	GCACTTAACCAAGTGCGCG
lmo1580-7	TCTACAATTTTTGCTCCCGC
lmo1580-8	GTAAAACTGCAGAACAAGTA
lmo2673-1	TAAGAGCTGCACTCGGTAGA
lmo2673-2b	TATACATGAATTTGTATCACCCTCTCAAAAGTTTTC
lmo2673-3b	GTGATACAAATTCATGTATAATGAAGGTATTG
lmo2673-4	CCAACAAGCGCGCAACGAATACC
lmo2673-5	AAAGAGACCCCCTTTTCCTC
lmo2673-6	TTTTTCTCCTCCTGCCGTAT
lmo2673-7	AGCATCCGTCACTAGCCCTG
lmo2673-8	GCTATCTTCGTAAGCAGTGA
*lmo0515*-forward	GATGGTTCAGAACCAGCAAA
*lmo0515*-reverse	GCTTTTTCTTCTAAGCTGCCAT
*lmo1580*-forward	GCAGTTGATGGATCCAAAGAA
*lmo1580*-reverse	TTTATCCGCCATGCTTGTATC
*lmo2673*-forward	ATAACGCGAAATTTTGAACCCG
*lmo2673*-reverse	CCAAGGTTTTGCGTGAACAA

For the generation of Δ*lmo0515* and Δ*lmo2673* the pCR2.1-TOPO® vector containing the flanking region of the respective gene was digested with *BamH*I, *Xho*I and *Nco*I (Fermentas) and inserted into pAUL-A [21], that was previously digested with *BamH*I and *Sal*I (Fermentas). The pCR2.1-TOPO® vector containing the flanking region of Δ*lmo1580* was digested with *Xba*I and *Sac*I (Fermentas) and ligated with pAUL-A, also previously digested with the same restriction endonucleases.

We designated resulting pAUL-A vectors containing the flanking regions of the respective *usp* genes, pCS1 for Δ*lmo0515*, pCS2 for Δ*lmo1580* and pCS3 for Δ*lmo2673*. These vectors were transformed into *E. coli* and were isolated, sequenced and subsequently electroporated into *L. monocytogenes* wild-type strain. Gene replacement was performed as previously described by Schaeferkordt et al. [21], [22]. To create a triple mutant (Δ*lmo0515*Δ*lmo1580*Δ*lmo2673*) for all three *usp* genes, gene replacement were carried out as described above starting with the electroporation of pCS2 into Δ*lmo0515*. Finally, pCS3 was electroporated into the resulting double mutant to generate the isogenic triple mutant (Δ*lmo0515*Δ*lmo1580*Δ*lmo2673*). The chromosomal deletion was confirmed by DNA sequencing of PCR products using primer 7 and 8 (Table 2).

The complementation of the Δ*lmo0515*, Δ*lmo1580* and Δ*lmo*2673 deletion mutants was carried out using the *L. monocytogenes* site specific phage integration vector pPL2 [23]. PCR products were generated using primer pairs 5 and 6 with Taq polymerase. The product was cloned into pCR 2.1-TOPO®. The pCR 2.1-TOPO® vector containing the *usp* gene region of *lmo0515* or *lmo2673* was digested with *BamH*I and *Xho*I (Fermentas) and for *lmo1580 with Xho*I and *Sac*I (Fermentas), respectively. The insert was ligated with pPL2, also previously digested with the same restriction endonucleases. Primer sequences used for the complementation of the deleted genes are listed in table 2.

### Acid stress assay

Single colonies of the wild type *L. monocytogenes* and its *usp* deletion mutants grown on BHI agar plates were used to inoculate 100 ml Erlenmeyer flasks containing 10 ml of BHI broth, followed by overnight incubation at 37°C with shaking (180 rpm, Unitron Infors). The overnight cultures were then subcultured (1∶50) in 40 ml of BHI and grown to an optical density at 600 nm (OD_600_) of 0.4 prior to our experiments. Then each culture was divided into two aliquots, 20 ml each and cells were harvested by centrifugation at 6,000×*g* for 15 min. at 37°C. Furthermore the supernatant was removed and cells were resuspended in a BHI broth that was acidified with a 6 N HCL solution (Sigma) to pH 2.5. These tubes were incubated with shaking (180 rpm) at 37°C. At time point zero (t = 0) and after 10, 20 and 30 minutes intervals samples were taken from each culture, serially diluted with phosphate-buffered saline (1×PBS) and plated on BHI agar plates. The colony forming units (CFU) were counted after overnight incubation at 37°C. The percentage of survivors was calculated by comparing the number of survivors in BHI (pH 2.5) after 10, 20 and 30 minutes with the number of cells in BHI (pH 2.5) at t = 0, respectively. Results are reported as means of data collected from three independent biological replicates.

### Hydrogen peroxide sensitivity assay

BHI broth was used to dilute a 3% hydrogen peroxide (H_2_O_2_) solution (Ottmar Fischer, Germany) to obtain 0.045% H_2_O_2_ final concentration.

Overnight cultures in 10 ml BHI broth (in 100 ml Erlenmeyer flasks) were prepared and were diluted 1∶50 in 20 ml fresh BHI broth or in 20 ml of different fresh BHI supplemented with 0.045% H_2_O_2_ (in 100 ml Erlenmeyer flasks) as mentioned before. The optical density (OD_600_) of the bacterial culture was measured at the indicated times after incubation at 37°C (180 rpm, Unitron, Infors).

### Macrophage survival assay

P388D1 murine macrophages, cultured in 24-well plates in RPMI (Life Technologies) supplemented with 10% fetal calf serum (FCS) and 1% nonessential amino acids (NEAs), were infected with 1×10^7^ bacteria to obtain an MOI of 10. After 30 min. RPMI was removed, and cells were washed twice with 1×PBS and incubated for 1 h in RPMI medium containing 50 µg/ml gentamicin. Macrophages were then washed three times with 1×PBS and lysed with ice-cold 0.2% Triton X-100 in H_2_O. The released bacteria were plated on BHI agar plates in appropriate dilutions and quantified after overnight incubation at 37°C.

For the determination of macrophage survival rates the CFU numbers in the inoculum of the wild-type and the mutants used to infect P388D1 were compared and a factor was calculated. The wild-type factor was set to 1 and depending on the CFU numbers of the mutant inoculums their factor was <1 or >1. For the calculation of the survival in % we set the CFU number of wild-type to 100% and percentage survival of the mutants was set in relation to wild-type.

### cDNA synthesis and quantitative Real-Time Polymerase Chain Reaction

Messenger RNA levels of *lmo0515*, *lmo1580* and *lmo2673* isolated from extracellular grown bacteria in BHI and intracellular replicating bacteria in murine macrophages as previously described [24] were assessed in *L. monocytogenes* wild-type strain using quantitative Real-Time-PCR (qRT-PCR). Complementary DNA (cDNA) was obtained from bacteria from each setting by reverese transcription of RNA isolated and purified using the RNeasy Mini kit (Qiagen). Triplicates were performed for each forward and reverse primer pair combination. Primers were purchased from Qiagen (Quantitect Primer Assay) (see Table 2).

Primers were diluted to 1 pmol/µl for further procedure and qRT-PCR was run (7900 HT Fast Real Time System, Applied Biosystems). 16sRNA was used as for normalization and calculation of relative expression.

Threshold cycle values (CT) of the tested genes were determined and normalized expression of each target gene was given as the ΔCT between the log2 transformed CT of the target gene and the log2 transformed CT of the internal control (ACTB). Log2 transformed gene expression levels (ΔCT) of each target gene for i5ntra- and extracellular bacteria were expressed as log2 differences from control ( = log2 ΔΔCT method). Data was acquired and analyzed with the SDS 2.3 and RQ-Manager 1.2, respectively.

### Animals and insects

Six to eight week-old female BALB/c mice, purchased from Harlan Winkelmann (Borchen, Germany), were kept at our breeding facilities in specific-pathogen-free conditions and used in all experiments. *Galleria mellonella* larvae, purchased from fauna topics (Marbach, Germany) were reared at 32°C in darkness and on an artificial diet (22% maize meal, 22% wheat germ, 11% dry yeast, 17.5% bee wax, 11% honey and 11% glycerine) prior to use. Last instar larvae, each weighing between 250 and 350 mg, were used in all experiments [20].

### Insect and animal models of infection

In all experiments, fresh cultures of bacteria, prepared from an overnight culture, were used. Briefly, bacteria were grown in Brain heart Infusion (BHI) at 37°C, harvested in the exponential growth phase and washed twice with 1×PBS. The pellet was resuspended in 1×PBS and the bacterial concentration was calibrated by optical absorption. Further dilutions were prepared in 1×PBS to obtain required numbers of bacteria for infection. The infection of *G. mellonella* was performed as previously described by Mukkerjee et al [20]. Briefly, the human pathogenic *L. monocytogenes* and its isogenic *usp* deletion mutants or complemeted strains were separately injected (10^6^ CFU/larva) into the hemocoel of the last instar larvae and the infection was monitored at 37°C.

In all experiments, fresh cultures of bacteria, prepared from an overnight culture, were used. Briefly, bacteria were grown in Brain heart Infusion (BHI) at 37°C, harvested in the exponential growth phase and washed twice with 1×PBS. The pellet was resuspended in 1×PBS and the bacterial concentration was calibrated by optical absorption. Further dilutions were prepared in 1×PBS to obtain required numbers of bacteria for infection.

Primary mice infections *in vivo* infection with *L. monocytogenes* wild-type, Δ*lmo0515*, Δ*lmo1580*, Δ*lmo2673* or Δ*lmo0515*Δ*lmo1580*Δ*lmo2673* were performed by an intravenous injection of viable bacteria in a volume of 0.2 ml of 1×PBS. Spleens and livers were harvested three days after infection. Bacterial growth in spleens and livers was determined by plating 10-fold serial dilutions of the organ homogenates on BHI agar plates. Colonies were counted after 24 h of incubation at 37°C.

### Ethics statement

This study was carried out in strict accordance with the regulation of the National Protection Animal Act (§7-9a Tierschutzgesetz). The protocol was approved by the local Committee on the Ethics of Animal Experiments (Regierungsbezirk Mittelhessen) and permission was given by the local authority (Regierungspraesidium Giessen, Permit Number: GI 15/5-Nr.63/2007).

### Statistical analysis

All experimental work was performed for a minimum of three times. Significant differences between two values were compared with a paired Student's *t*-test. Values were considered significantly different when *P*<0.05.

## Results

### Comparative genomic analysis and promoter prediction

**Figure 1:**
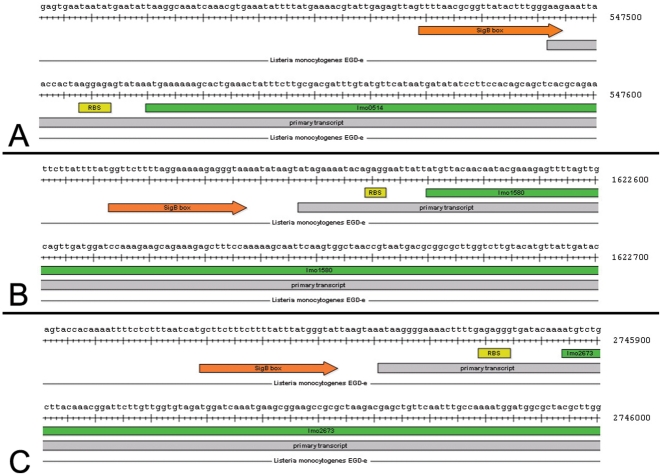
Transcriptional overview of chromosomal loci of *lmo0515* (A), *lmo1580* (B), and *lmo2673* (C) derived from sequencing data of *L. monocytogenes* (data not shown). σ^B^ boxes are depicted as orange arrows, the primary transcripts from their start sites by grey bars, the ribosome binding site as a yellow box and the respective coding sequence from their start sites by green bars.

All three Usps of *L. monocytogenes* harbor an UspA domain based on the Pfam analysis (data not shown), and thus represent paralogues of the UspA family of *E. coli* [1]. The structure and function of UspA is highly conserved among several bacteria and eukaryotic organisms. In line with this, comparative analysis in 19 listerial strains including all serotypes using GECO [25] (cut-off protein identity 80%, 90%) revealed a common chromosomal location for all three *usp* genes (Figure S1, S2, S3). Previously identified regulatory RNAs [26] were not located in the flanking regions of the *usp* genes. Furthermore, promoter box analysis of the upstream regions indicated that all *usp* genes habour a σ^B^ box upstream of their trancriptional start sites (Figure 1).

### Universal stress proteins contribute to extracellular survival of *L. monocytogenes* at low pH condition

**Figure 2:**
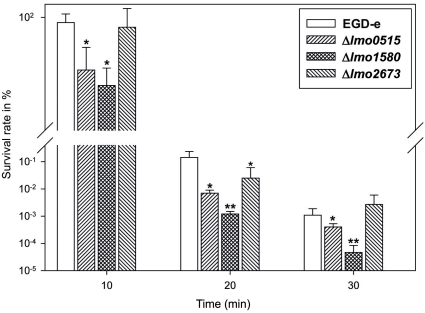
Effect of low acidic condition (pH = 2.5) on survival of *usp* deletion mutants compared to *L. monocytogenes* EGD-e wild-type. Δl*mo0515* and Δ*lmo1580* display decreased growth at all observations, while impaired resistance in Δ*lmo2673* was visible only at 20 min post challenge. Statistically significant differences were identified using a two-tailed Student *t* test. (*, *p*<0.05 and **, *p*<0.005).

*L. monocytogenes* is able to resist and survive at low pH conditions, which may occur in acidic food, in the gastric milieu and within the macrophage phagosome [27], [28]. In order to assess the role of listerial Usps at low pH environment we challenged exponentially grown *L. monocytogenes* and its isogenic *usp* deletion mutants (Δ*lmo0515*, Δ*lmo1580* and Δ*lmo2673*) with acidic BHI-medium (pH = 2.5) and determined the percentage of CFU at 10, 20 and 30 min after inoculation (Figure 2).

The CFU counts of both, Δ*lmo0515* and Δ*lmo1580* were significantly lower compared to wild-type bacteria at each time point. We observed no significant difference in survival rate between wild-type and Δ*lmo2673*. Δ*lmo1580* was most susceptible to an increase in acid over the time course which was reflected by a more than 100 fold lower survival rate of the culture as compared to the wild-type strain.

### Universal stress proteins are crucial for oxidative stress resistance of *L. monocytogenes*

*L. monocytogenes* is known to be resistant to oxidative stress, occurring in the macrophage phagosome, where reactive oxygen species (ROS), such as H_2_O_2_ are produced to kill engulfed pathogens [29].

**Figure 3:**
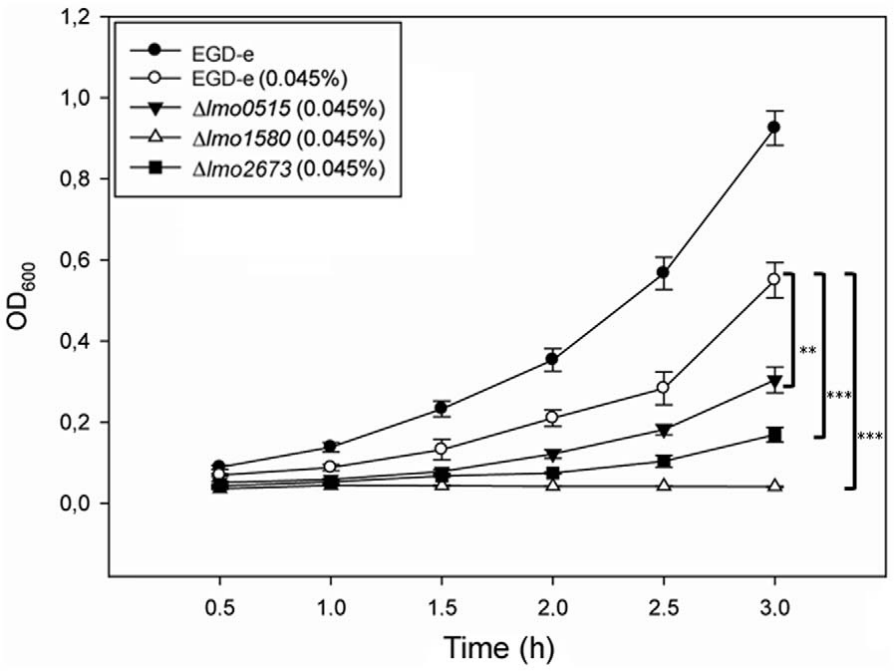
Survival of *L. monocytogenes* EGD-e wild-type and *usp* deletion mutants after exposure to H_2_O_2_. As appreciable, resistance of all deletion mutants was strongly impaired, resulting in decreased growth as compared to wild-type strain. Statistically significant differences were identified using a two-tailed Student *t* test. (*, *p*<0.05; **, *p*<0.005, and ***, *p*<0.0005).

To elucidate the role of listerial Usps in response to oxidative stress, we determined the survival of Δ*lmo0515*, Δ*lmo1580* and Δ*lmo2673* using optical density measurement in BHI medium supplemented with H_2_O_2_ following a 3 h incubation period (Figure 3).

Exposure to H_2_O_2_ significantly impaired the growth of the mutants compared to the wild-type strain (OD_600_ = 0.55). While growth attenuation was moderate in Δ*lmo0515* (OD_600_ = 0.3), a dramatic effect was seen for the *lmo2673* mutant (OD_600_ = 0.16). The strongest effect was observed in Δ*lmo1580* with barely any growth detectable at any observation point. These results indicate a gradual order in susceptibility to oxidative stress in the three *usp* mutants as follows: Δ*lmo1580*>Δ*lmo2673*>Δ*lmo0515*.

### Survival attenuation of Δ*lmo1580* and Δ*lmo2673* in murine macrophages

Macrophages play a key role in the phagocytosis and intracellular killing of *L. monocytogenes* by generating ROS and acidic conditions in the phagosome. To examine the impact of Usps for intracellular survival, we determined the number of CFU isolated from P388D1 murine macrophages upon infection with either wild-type strain or the *usp* deletion mutants [30].

**Figure 4:**
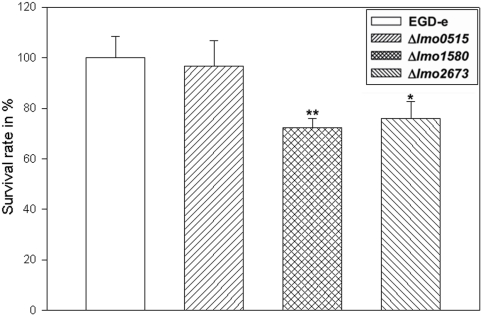
The intracellular survival of *L. monocytogenes* EGD-e wild-type and *usp* deletion mutants was assessed in murine macrophages according to [**30**]. Intracellular bacteria burden was decreased in macrophages inoculated with Δ*lmo1580* and Δ*lmo2673*, but no effect was visible for Δ*lmo0515*. Statistically significant differences were identified using a two-tailed Student *t* test. (*, *p*<0.05 and **, *p*<0.005).

**Figure 5:**
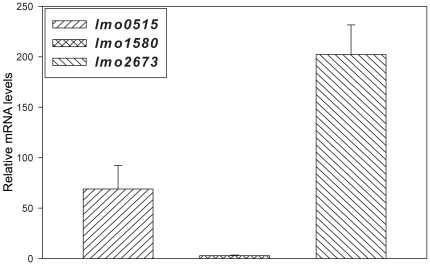
qRT-PCR analysis of *lmo0515*, *lmo1580* and *lmo2673* expressed in *L. monocytogenes* EGD-e. The graph displays the relative expression of the respective *usp* gene in the intracellular vs. extracellular setting. High fold changes indicate that the particular gene was higher expressed in intracellularly localized bacteria as compared to expression in bacteria that were grown in BHI. The expression of *lmo0515* and *lmo2673* is strongly enhanced in intracellular pathogens, while levels of *lmo1580* remain relatively constant. This is consistent with the observation made in this study, which shows a strong dependence on *lmo2673* and *lmo0515* in the murine infection model experiments as compared to extracellular *in vitro* challenges, while *lmo1580* seems to play an important role in both conditions.

In agreement with our results at low pH condition and the oxidative stress response, the Δ*lmo1580* deletion had the strongest effect on intracellular listerial survival. Compared to the wild-type strain, only 72% of Δ*lmo1580* were able to resist the hostile macrophage environment. Δ*lmo2673* exhibited similar survival attenuation with 76% viable bacteria compared to wild-type strain. No significant effect on intracellular survival was detected in the Δ*lmo0515* mutant (Figure 4). To confirm that the *usp* genes are intracellular induced we appllied quantitative RT-PCR. The analysis showed that *usp* transcripts obtained from intracellular versus extracellular grown bacteria displayed a strong induction of *usp* genes (Figure 5).

### Decreased virulence of *usp* mutants in an invertebrate *in vivo* model

*G. mellonella* is currently a well established model host for studying the virulence attributes of the human pathogenic *L. monocytogenes* [20]. In this work, we used this invertebrate model to evaluate the pathogenicity of listerial *usp* deletion mutants. *G. mellonella* larvae were infected with pathogenic EGD-e and its isogenic *usp* mutants namely Δ*lmo1580*, Δ*lmo2673* and Δ*lmo0515* respectively. Survival rate of the infected larvae was determined at 37°C for over a period of 7 days.

**Figure 6:**
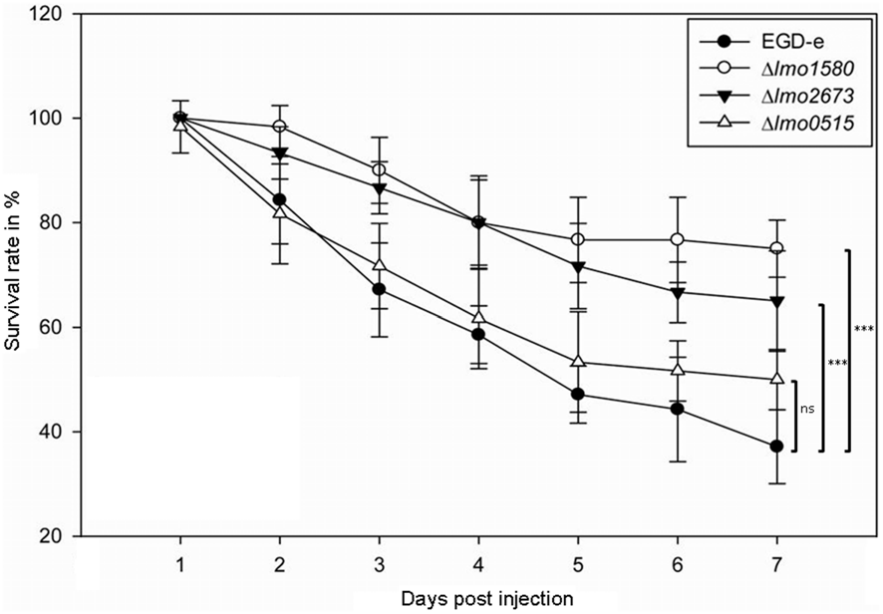
Survival of *G. mellonella* larvae after inoculation with *usp* deletion mutants. As compared to *L. monocytogenes* EGD-e wild-type, all *usp* deletion mutants exhibited impaired virulence, resulting in higher survival rates of *G. mellonella* larvae. Strongest effects were observed for Δ*lmo1580 and* Δ*lmo2673*, while deletion of *lmo0515* was not associated with significant increase in survival of *G. mellonella*. Results represent mean values of at least three independent experiments with a total of 80 larvae per treatment. Statistically significant differences were identified using a two-tailed Student *t* test. (***, *p*<0.0005 and ns-not significant).

Attenuated virulence associated with higher survival rates of larvae (Figure 6) were observed in Δ*lmo1580*, Δ*lmo2673* and Δ*lmo0515* (75%, 65% and 50% respectively) as compared to wild-type infection (37%). The difference between Δ*lmo0515* and wild-type did however not reach statistical significance. As observed previously with intracellular growth in macrophages, the Δ*lmo1580* mutation had the strongest effect on the pathogenicity, exhibiting nearly avirulent characteristics. A similar result was obtained for the triple mutant Δ*lmo0515*Δ*lmo1580*Δ*lmo2673* (Figure S4). To confirm that the deletion of *usp* genes is responsible for the effects in invertebrate infections we complemented the single *usp* mutants. Complementation restored the ability to kill insect larvae to levels comparable to the wild-type bacteria (Figure S5).

We observed no differences in growth of *usp* mutants and wild-type strain when cultured in BHI (data not shown) which excludes the possibility that varying numbers of inoculum CFUs of *usp* deletion mutants affected the survival of insect larvae.

### Growth and survival of *Listeria usp* deletion mutants is profoundly impaired in a murine infection model

Although several studies have investigated the role of Usps in different bacteria, an important role of Usps *in vivo* has only been shown for the regulation of growth in *S. typhimurium* [12] and *M. tuberculosis* [6]. To elucidate the influence of listerial Usps on the growth and survival *in vivo*, we injected BALB/c mice with three different *usp* deletion mutants or wild-type and determined the number of bacteria isolated from the liver.

**Figure 7:**
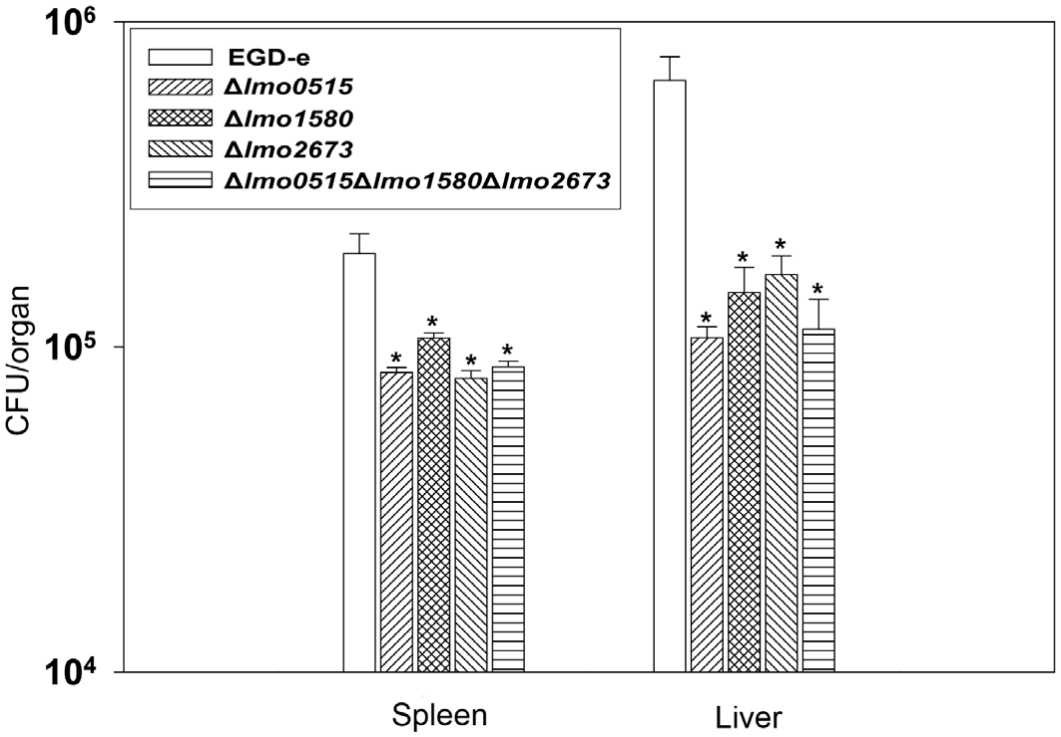
Survival rates of intravenously inoculated *L. monocytogenes* EGD-e or *usp* deletion mutants in mice livers and spleens. Survival of mutant bacteria are impaired in both organs, reflecting a role of Usps in listerial resistance and growth *in vivo*. Statistically significant differences were identified using a two-tailed Student *t* test. (*, *p*<0.05).

Remarkably, the number of CFU of all mutants compared to wild-type was dramatically diminished in this mouse model (Figure 7). The number of viable bacteria lacking the respective *usp* gene was significantly lower in livers and spleens of infected mice. In this setting, growth of Δ*lmo0515* was reduced to 22% in spleen and 32% in liver as compared to wild-type strain. The number of Δ*lmo2673* was reduced to 68% and 60%, and the Δ*lmo1580* count declined to 38% and 68%, respectively. The isogenic *usp* triple mutant showed comparable results as the single *usp* mutants.

## Discussion

*L. monocytogenes* is capable of withstanding a variety of stressors encountered in nature and within infected host cells [18]. The ability to rapidly adjust to the changing environment is essential for its pathogenic lifestyle [18]. In this study, we provide strong evidence that universal stress proteins (Usps) are highly conserved among *Listeriae* and are important for resistance and growth of *L. monocytogenes* by investigating *usp* deletion mutants exposed to different stress conditions both *in vitr*o and *in vivo*.

Universal stress proteins were shown to be important in response to stress in gram-negative bacteria, including *E. coli* [31], *S. typhimurium* [12] and *Azospirillium brasilense* [32]. However, the role of these proteins in gram-positive bacteria is not as well delineated. We have previously revealed an induction of three genes, *lmo0515*, *lmo2673* and *lmo1580* in *L. monocytogenes* in response to heat shock and acid stress [33], [34]. In accordance with effects described for *E. coli* lacking the *uspA* gene, *L. monocytogenes usp* deletion mutations investigated in this study displayed impaired capability to resist exposure to H_2_O_2_ and low pH conditions. Variable susceptibility to stress conditions between deletion mutants may represent a non-redundant role of Usps in stress responses. Similar observations were made for *E. coli* and addressed in a recent study testing several *usp* deletion mutants [31]. Nachin et al. uncovered that the UspA proteins differ in their responses to oxidative stress and DNA damaging agents and defined similarities as well as differences in expression pattern based on their biological role in stress response [31]. Thus, certain environmental changes may not require the induction of all *usp* genes, but allow adjustment depending on the specialized role of a particular Usp.

Generally, *usp* genes are expressed in monocistronic units and the production of UspA seems to be primarily regulated at the level of transcription initiation [1]. We and others have previously reported an important role for σ^B^, a transcription factor that controls an inducible regulon in *L. monocytogenes* induced following challenge to different stress conditions, such as acids, high osmolarity or carbohydrate starvation [17], [34], [35]. As previously reported, a single σ^B^ promoter has been identified for *lmo1580*; and promoter analysis revealed σ^B^ boxes for *lmo2673* and *lmo0515*. Thus, it appears likely that σ^B^ possesses a major role in stress induced transcriptional regulation of *usp* genes in *L. monocytogenes*. Furthermore, phenolic acids and fructose-6-phosphate were shown to influence *usp* gene expression in *Lactobacillus plantarum* and *E* coli, respectively. *L. monocytogenes* mobilizes a large set of metabolic genes in response to stress [24]. Especially, genes of the carbohydrate metabolism are strongly expressed. Possible effects of metabolic constituents on *usp* gene regulation may therefore not be excluded and require further investigation. [15], [36].

In this regard, the temporal and coordinated activities of different Usps of *L. monocytogenes* may enable the bacteria to survive in acid-rich niches such as in the upper intestinal tract or mediate resistance to oxidative stress and bile acids in the spleen and liver.

Following ingestion by phagocytic cells, *Listeriae* are faced with the hostile environment in the phagosome [18]. As shown previously, virulence and intracellular growth adaption of *L. monocytogenes* depends on a concerted gene expression program including the up-regulation of universal stress proteins [24], [37]. In this study, we demonstrate that survival of *Listeriae* in murine macrophages is dependent on the presence of Usps. This was reflected by decreased virulence, resulting in a significant reduction of bacteria lacking the respective *usp* genes. In addition we observed intracellular transcriptional induction of all three *usp* genes which supports the evidence that the particular stress proteins are involved in the intracellular pathogenic lifestyle of *L. monocytogenes*.

The impact of Usps in infection has only been examined for a few bacteria and their role, if any, in gram-positive pathogens has not been described [6], [12], [31], [37]. For this reason, we further characterized the effect of listerial Usps in *in vivo* infection models. First we tested their effects on the viability of *G. mellonella* larvae, a recently described alternative infection model for *L. monocytogenes* [20]. Lack of *usp* genes resulted in improved survival of infected larvae as compared to wild-type infection. In accordance with our *in vitro* results, Δ*lmo1580* was nearly avirulent demonstrating its importance in pathogenicity of *L. monocytogenes*. As also demonstrated here, Usps contribute strongly to the survival and proliferation of these bacteria in livers and spleens of infected mice, while no major differences were apparent when comparing both organs. We have also constructed a triple mutant to investigate the cooperative nature of the three *usp* genes. We could not observe any synergistic effect for the *usp* triple mutant in mice experiments, but in the invertebrate infection model the isogenic triple mutant indicated a similar survival rate for larvae infected with Δ*lmo1580*. Thus, we suspect that Δ*lmo1580* might be the major player among the three *usp* genes involved in pathogenicity.

Previously, Usps were implicated in the resistance to antibiotics [38], biofilm formation by *P. gingivalis* [10] and survival of *P. aeruginosa* under anaerobic conditions [7]. In this study however, we did not identify a significant impact of *usp* gene deletion on biofilm formation or altered resistance under anaerobic/microaerophilic conditions and in response to treatment with several antibiotics (data not shown).

In conclusion, we have identified an important role of three universal stress proteins in resistance and survival of *L. monocytogenes* in response to low pH conditions and oxidative stress as well as intracellular survival within macrophages. We subsequently confirmed our observations in two *in vivo* infection models and show for the first time that universal stress proteins are important for survival and growth in the vertebrate and invertebrate hosts. Finally, we propose the designation of genes for *lmo0515*, *lmo1580* and *lmo2673 as uspL-1* (universal stress protein *Listeria*), *uspL-2* and *uspL-3*, respectively in the species *L. monocytogenes*.

## Supporting Information

Figure S1 Comparative analysis of flanking regions of listerial universal stress protein *lmo0515* among 19 *L. monocytogenes* species including all known serotypes.
(PDF)

Figure S2 Comparative analysis of flanking regions of listerial universal stress protein *lmo1580* among 19 *L. monocytogenes* species including all known serotypes.
(PDF)

Figure S3 Comparative analysis of flanking regions of listerial universal stress protein *lmo2673* among 19 *L. monocytogenes* species including all known serotypes.
(PDF)

Figure S4 Survival of *G. mellonella* larvae after inoculation with *usp* deletion mutants. As compared to *L. monocytogenes* EGD-e wild-type, triple deletion mutant of *lmo1580*, *lmo2673* and *lmo0515* exhibited impaired virulence similar to the single gene deletion mutant Δ*lmo1580*, resulting in significant higher survival rates of *G. mellonella* larvae. Results represent mean values of at least three independent experiments and each repetition contained 30 larvae per treatment. Statistically significant differences were identified using a two-tailed Student *t* test. (***, *p*<0.005).
(TIF)

Figure S5 Complemetation of *lmo1580*, *lmo2673* and *lmo0515* into their respective isogenic deletion mutants Δ*lmo1580*, Δ*lmo2673* and Δ*lmo0515* resulted in induced virulence. Artificial introduction of the individual *usp* genes *lmo1580*, *lmo2673* and *lmo0515* into their respective isogenic deletion mutants Δ*lmo1580*, Δ*lmo2673* and Δ*lmo0515* resulted in an increase of virulence similar to the wild-type EGD-e. Results represent means of at least three independent determinations ± standard deviations. Each repetition contained 20 larvae per treatment.
(TIF)
