# Supplementary figures and images for: Universal Stress Proteins Are Important for Oxidative and Acid Stress Resistance and Growth of Listeria monocytogenes EGD-e In Vitro and In Vivo
Source: PLoS One. 2011 Sep 30;6(9):e24965. doi: 10.1371/journal.pone.0024965 (PMC3184099; doi:10.1371/journal.pone.0024965)

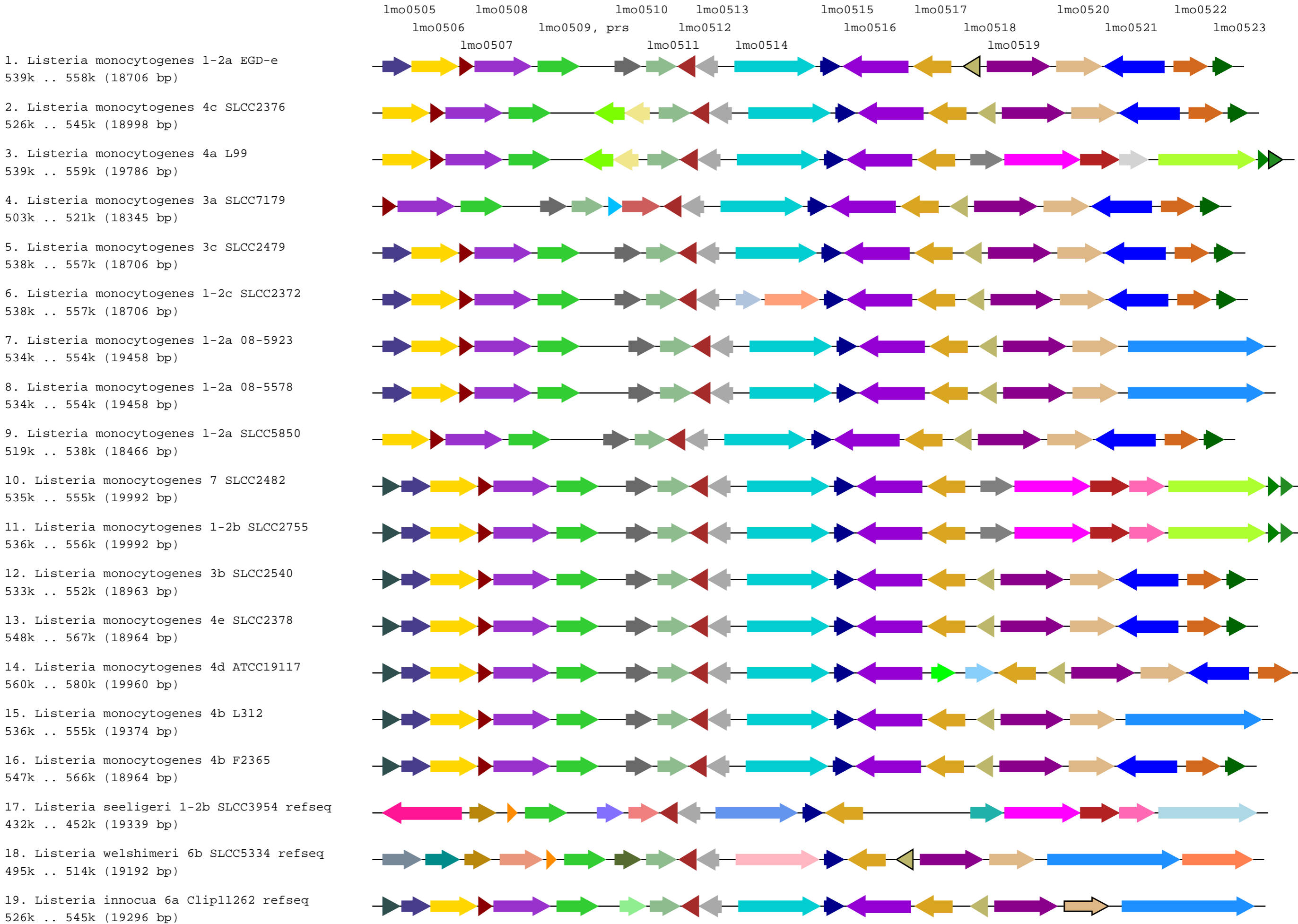

Supplement: Figure S1 — Comparative analysis of flanking regions of listerial universal stress protein lmo0515 among 19 L. monocytogenes species including all known serotypes. (PDF) [file pone.0024965.s001.pdf]

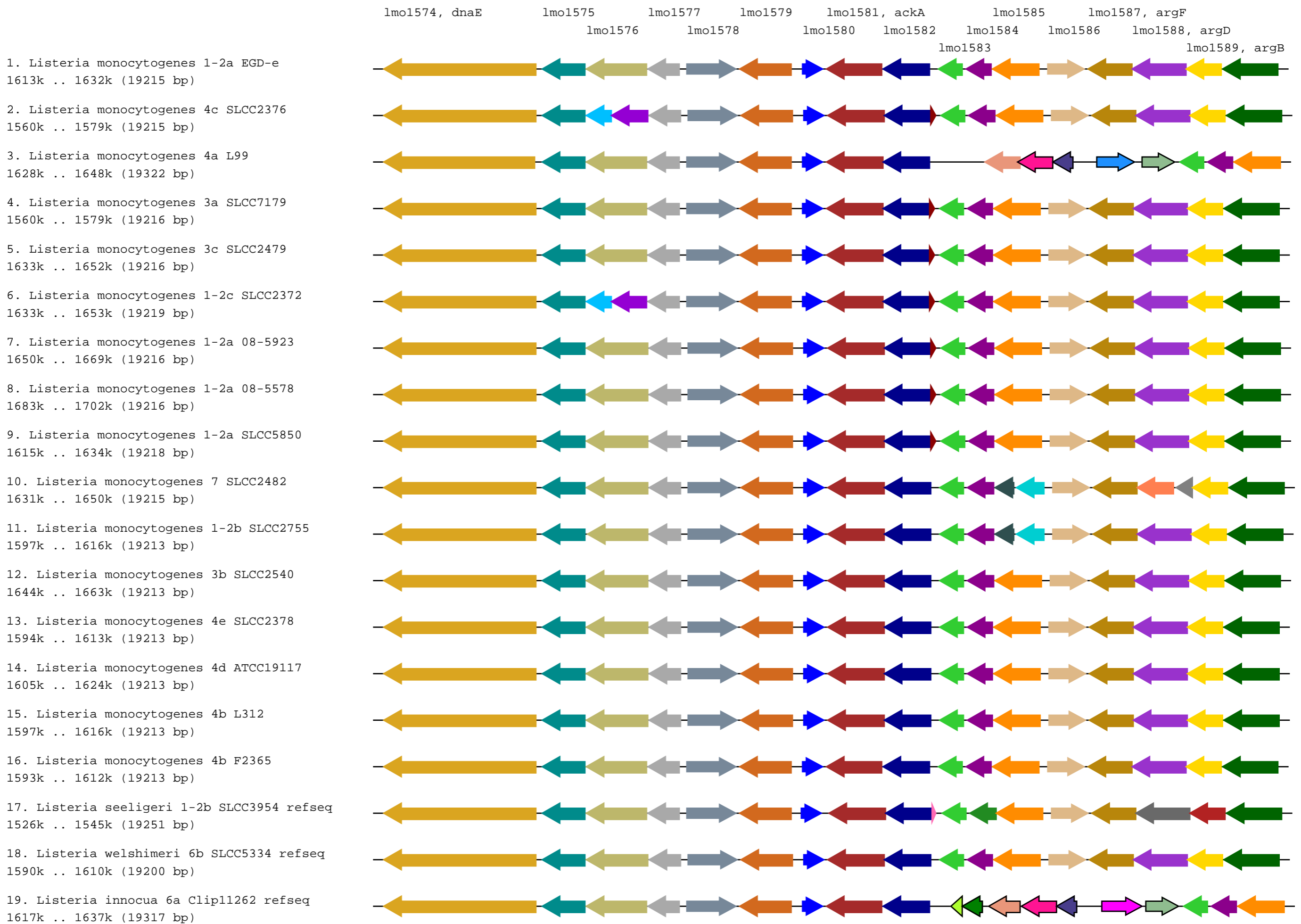

Supplement: Figure S2 — Comparative analysis of flanking regions of listerial universal stress protein lmo1580 among 19 L. monocytogenes species including all known serotypes. (PDF) [file pone.0024965.s002.pdf]

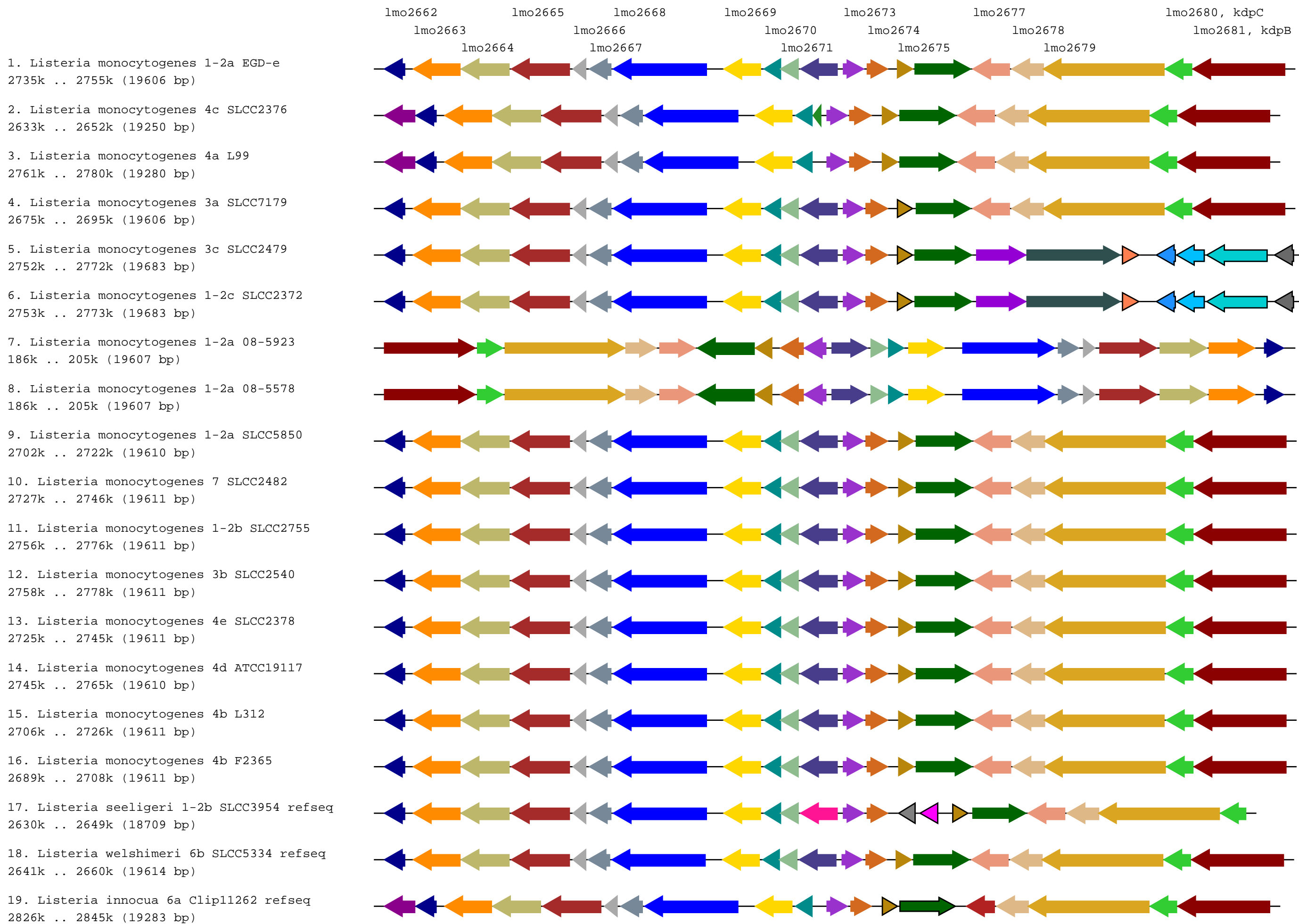

Supplement: Figure S3 — Comparative analysis of flanking regions of listerial universal stress protein lmo2673 among 19 L. monocytogenes species including all known serotypes. (PDF) [file pone.0024965.s003.pdf]

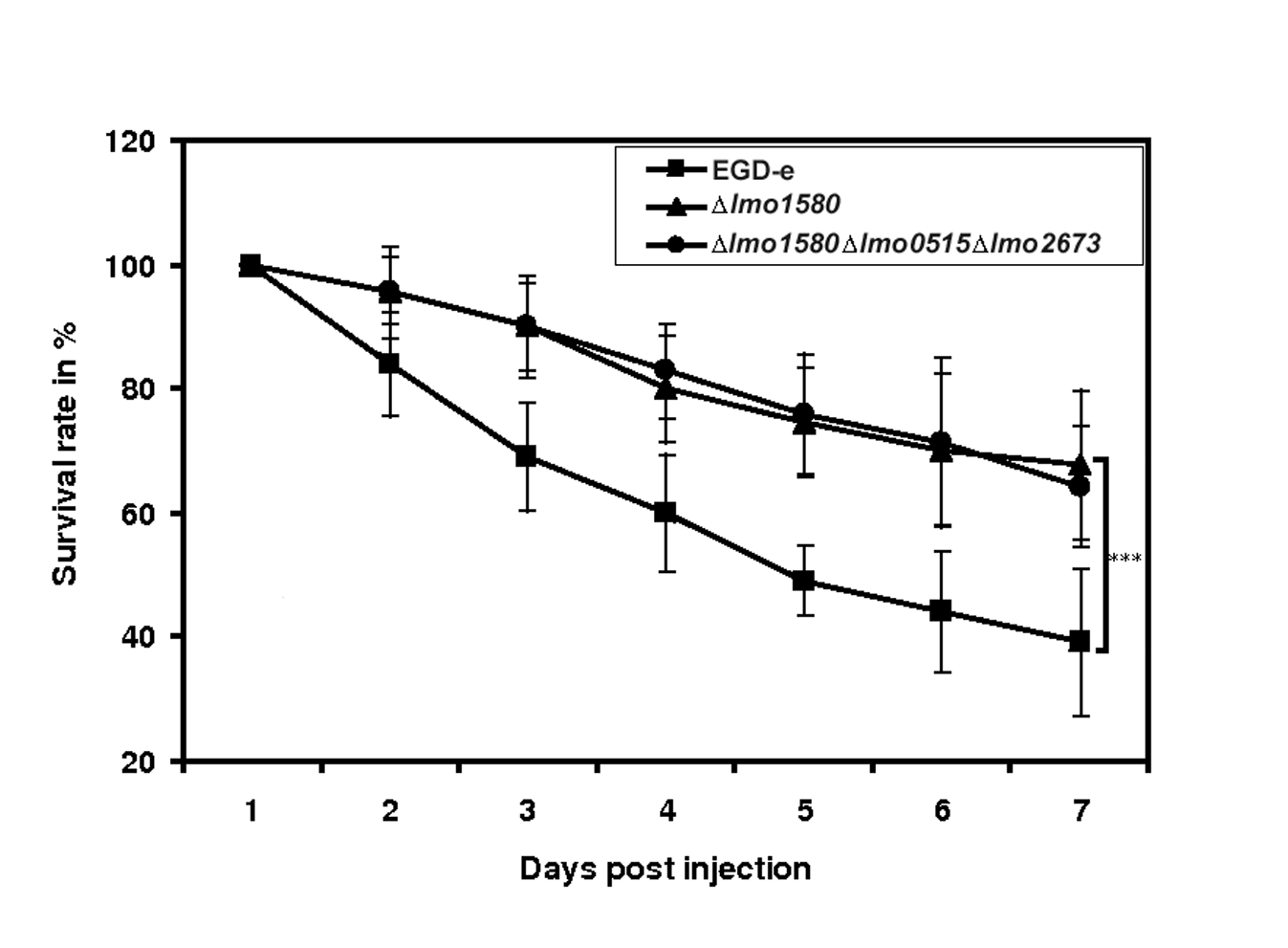

Supplement: Figure S4 — Survival of G. mellonella larvae after inoculation with usp deletion mutants. As compared to L. monocytogenes EGD-e wild-type, triple deletion mutant of lmo1580, lmo2673 and lmo0515 exhibited impaired virulence similar to the single gene deletion mutant Δlmo1580, resulting in significant higher survival rates of G. mellonella larvae. Results represent mean values of at least three independent experiments and each repetition contained 30 larvae per treatment. Statistically significant differences were identified using a two-tailed Student t test. (***, p<0.005). (TIF) [file pone.0024965.s004.tif]

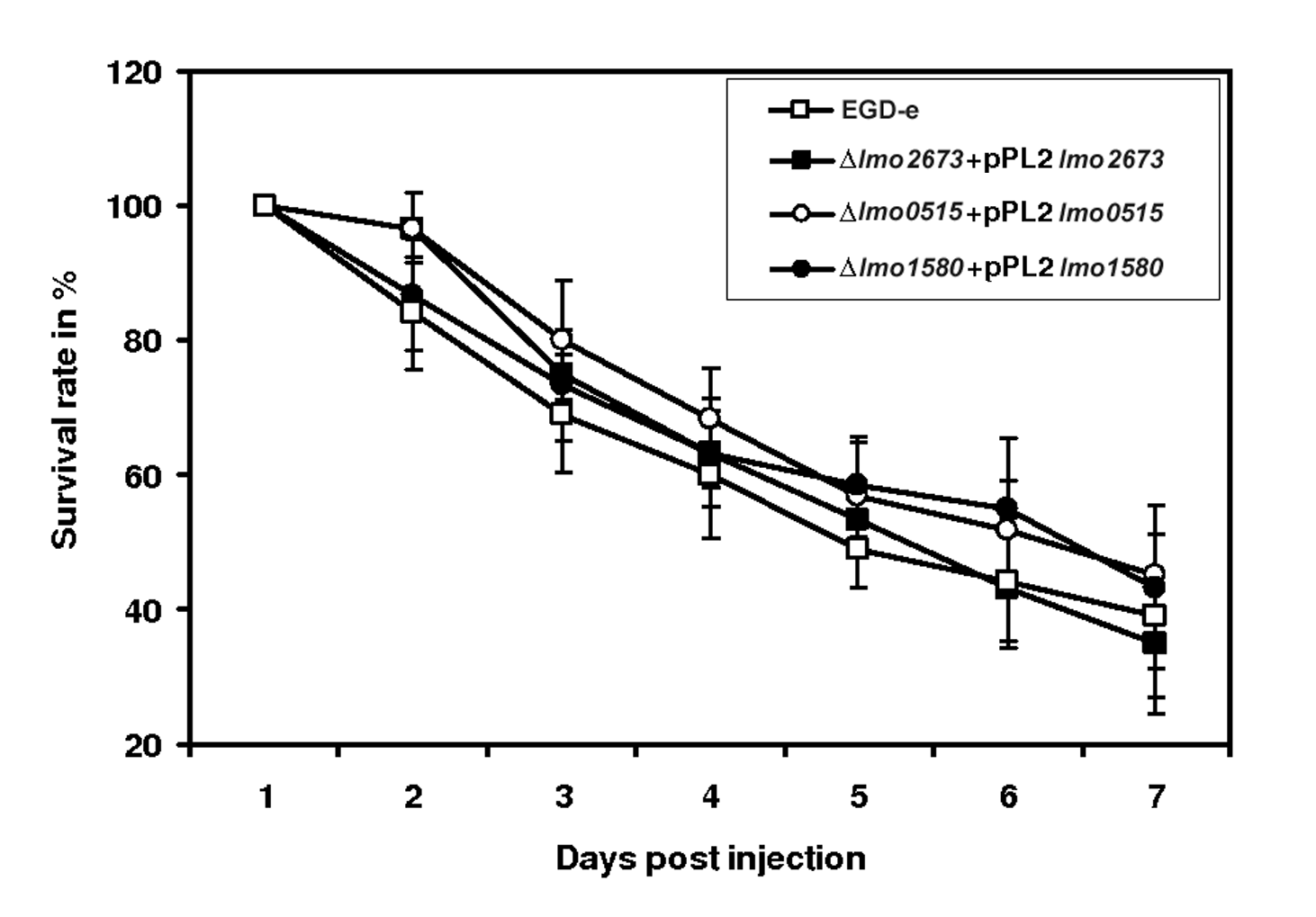

Supplement: Figure S5 — Complemetation of lmo1580, lmo2673 and lmo0515 into their respective isogenic deletion mutants Δlmo1580, Δlmo2673 and Δlmo0515 resulted in induced virulence. Artificial introduction of the individual usp genes lmo1580, lmo2673 and lmo0515 into their respective isogenic deletion mutants Δlmo1580, Δlmo2673 and Δlmo0515 resulted in an increase of virulence similar to the wild-type EGD-e. Results represent means of at least three independent determinations ± standard deviations. Each repetition contained 20 larvae per treatment. (TIF) [file pone.0024965.s005.tif]
